# Reverse Phenotyping: Addressing Refractory Seizures From an Endocrine Perspective

**DOI:** 10.7759/cureus.75146

**Published:** 2024-12-05

**Authors:** Shijiya Sherin, Dhanya Soodhana, Smilu Mohanlal, Divya Pachat

**Affiliations:** 1 Department of Pediatrics, Aster Malabar Institute of Medical Sciences, Kozhikode, IND; 2 Pediatric and Adolescent Endocrinology, Aster Malabar Institute of Medical Sciences, Kozhikode, IND; 3 Neurology, Aster Malabar Institute of Medical Sciences, Kozhikode, IND; 4 Clinical Genetics, Aster Malabar Institute of Medical Sciences, Kozhikode, IND

**Keywords:** diazoxide, epilepsy, glud1, hyperinsulinism-hyperammonemia (hi/ha) syndrome, neurodevelopmental disorders

## Abstract

Neonatal hypoglycemia (NH) is a common abnormality in newborns, posing significant morbidity risks. Prompt diagnosis and treatment are vital to mitigate brain damage and enhance outcomes. Congenital hyperinsulinemia (CHI) is a leading cause of recurrent hypoglycemia in infants, often stemming from genetic mutations such as in the *GLUD1* gene, manifesting as hyperinsulinism-hyperammonemia syndrome (HI/HA).

We present a case of a 2-year-old girl with refractory epilepsy, later identified as HI/HA, whose paroxysmal episodes mimicked multiple seizure types. Genetic testing revealed a heterozygous pathogenic mutation in exon 2 of the *GLUD1* gene. Treatment with diazoxide significantly improved blood sugar levels and achieved effective seizure control.

Our case underscores the significance of considering metabolic etiologies like hyperinsulinemic hypoglycemia in children with seizures resistant to standard antiepileptic drugs. Early recognition, genetic testing, and targeted therapy are pivotal for achieving seizure control and optimizing patient outcomes.

## Introduction

Congenital hyperinsulinism (CHI) encompasses a spectrum of disorders arising from mutations in genes regulating insulin secretion, resulting in chronic hyperinsulinemic hypoglycemia. Neurological impairment may arise from hypoglycemic episodes, especially in the neonatal period, depending on their severity and duration. Despite the straightforwardness of blood sugar assessment, signs of hypoglycemia can often be nonspecific [1]. Seizures due to low blood glucose levels (less than 2 mmol/l) can impede development, induce motor and learning disabilities, and in severe cases, lead to death. While individual thresholds must be considered, clinically significant hypoglycemia is defined as a plasma glucose concentration low enough to induce symptoms and/or evidence of impaired brain function [2].

**Figure 1:**
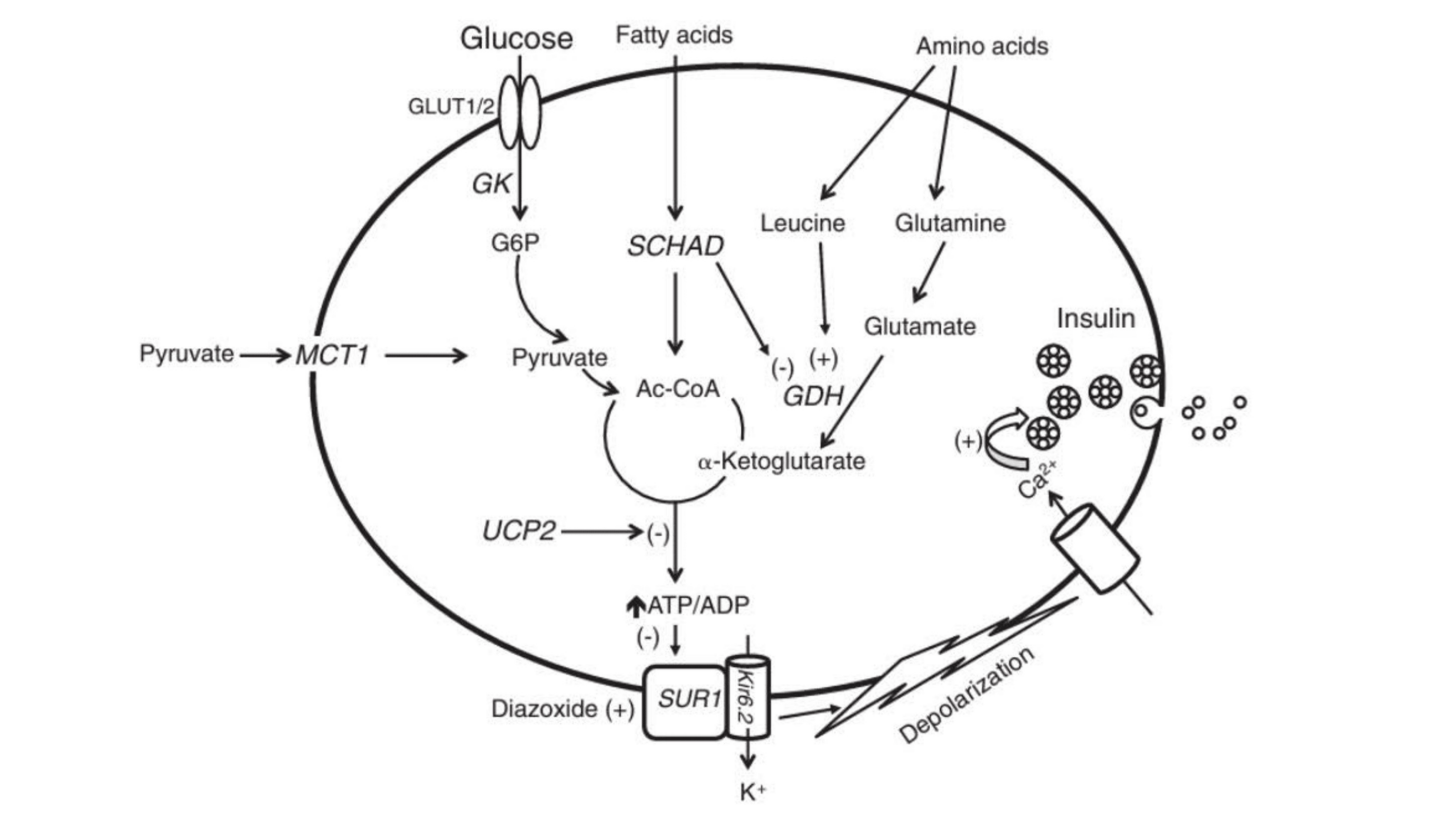
Common mutations associated with CHI (1) ATP-gated K+ channel (KATP) encoded by ABCC8 and KCNJ11; (2) Glutamate dehydrogenase (GDH) encoded by GLUD1; (3) Glucokinase (GCK) encoded by GCK gene; (4) L-3-hyroxyacyl-coenzyme A dehydrogenase (HADH) encoded by HADH; (5) Hepatocyte Nuclear Factor 4α (HNF4α) encoded by HNF4A gene; (6) The monocarboxylate transporter (MCT1) encoded by SLC16A1; (7) Uncoupling Protein 2 (UCP2) CHI: congenital hyperinsulinemia; SCHAD: short-chain-hydroxyacyl-CoA dehydrogenase The image is drawn by the authors of this article.

With an incidence of 1 in 200,000, hyperinsulinism-hyperammonemia syndrome ranks as the second most prevalent cause of hyperinsulinism in infancy, attributed to an activating heterozygous mutation in the *GLUD1* gene, encoding the intra-mitochondrial enzyme glutamate dehydrogenase (GDH) [3]. GDH is expressed significantly in the liver, kidney, pancreatic β-cells, and brain. It facilitates the oxidative deamination of glutamate to α-ketoglutarate and ammonia. α-ketoglutarate enters the tricarboxylic acid cycle in pancreatic β-cells, triggering insulin exocytosis (Figure 1). Leucine allosterically activates GDH, while GTP inhibits it. Mutations activating the *GLUD1* gene reduce the enzyme’s susceptibility to GTP and ATP-induced allosteric inhibition. Loss of GTP-induced inhibition by leucine enhances glutamate oxidation to α-ketoglutarate [4]. Clinically, leucine sensitivity manifests as hypoglycemic symptoms typically between 4 and 6 months of age, triggered by fasting or high-protein meals, often accompanied by elevated serum ammonia [5]. The severity of hypoglycemia can vary.

The risk of brain damage appears to stem more from delays in detection and treatment rather than the genetic abnormality itself and may thus be preventable. Our case illustrates the link between seizures in hyperinsulinism-hyperammonemia (HI/HA) syndrome, which can complicate diagnosis and delay intervention.

## Case presentation

A 2-year-old girl, born as the fifth child of non-consanguineous parents, presented at 7 months with a history of seizures. She was born at term with a birth weight of 2.6 kg and experienced asymptomatic hypoglycemia in the perinatal period, which resolved with oral feeds. From the postnatal records, a documented blood glucose value of 40 mg/dl was noted. Newborn screening was not done. There is a family history of one intrauterine death and two children with cyanotic congenital heart disease. One child died at 2.5 years, and the other with ambiguous genitalia is currently 6 years old boy and doing well.

Common mutations associated with CHI are demonstrated in Figure 1.

At 9 months, the child presented to our hospital with new-onset seizures characterized by focal eye deviation and clonic jerking, occurring three times daily. She also exhibited motor delay, attaining neck control at 6 months. Initial investigations including electroencephalogram and brain magnetic resonance imaging were unremarkable. Metabolic workup was negative, and she was initiated on antiepileptic medications (valproate, zonisamide). The elevated ammonia levels were probably attributed to the child being on valproate or delayed processing. Subsequent follow-up revealed polymorphic seizures with episodes resembling alternating hemiplegia. In view of refractoriness, multiple antiepileptic drugs including phenobarbitone were added. During the paroxysmal episodes, hypoglycemia was documented (random blood sugar (RBS) 38 mg/dl) once and was considered secondary to seizure.

**Figure 2:**
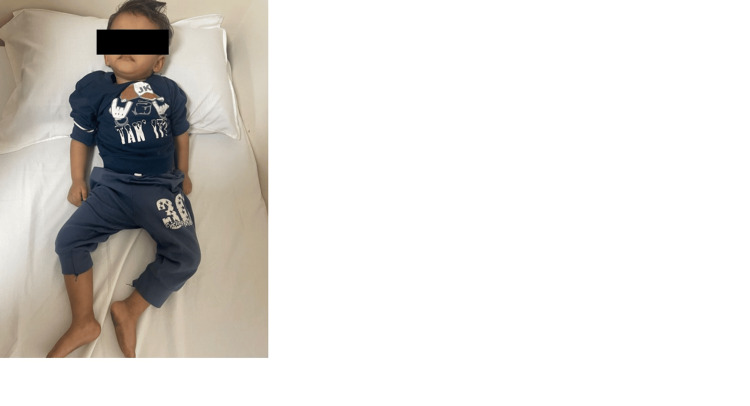
Upon examination, the child’s weight was 8.2 kg (-3.16 z score), height 80 cm (-2.24 z score), and head circumference 44 cm (-2.47 z score), with no syndromic features.

Due to the unknown etiology of refractory seizures, genetic testing was pursued. Whole exome sequencing revealed a heterozygous pathogenic variant in exon II of the *GLUD1 *gene located on chromosome 10q23.3, suggestive of hyperinsulinemic hypoglycemia. Upon examination, the child's weight was 8.2 kg (-3.16 z score), height 80 cm (-2.24 z score), and noted to have microcephaly-head circumference 44 cm (-2.47 z score), with no syndromic features (Figure 2). Random blood sugar was 44 mg/dl at the time of investigation. During hypoglycemia, insulin was 16.72 µIU/ml (<2 µIU/ml), C-peptide was 3.8 ng/ml (normal range 0.9-1.8 ng/ml), blood ketone level was 0 mmol/l and plasma ammonia was elevated at 253 µmol/l (normal range 16-60 µmol/l), indicating hypoketotic hypoglycemia consistent with hyperinsulinism with hyperammonemia. No temporal association between protein-rich meals and seizure episodes was observed.

**Figure 3:**
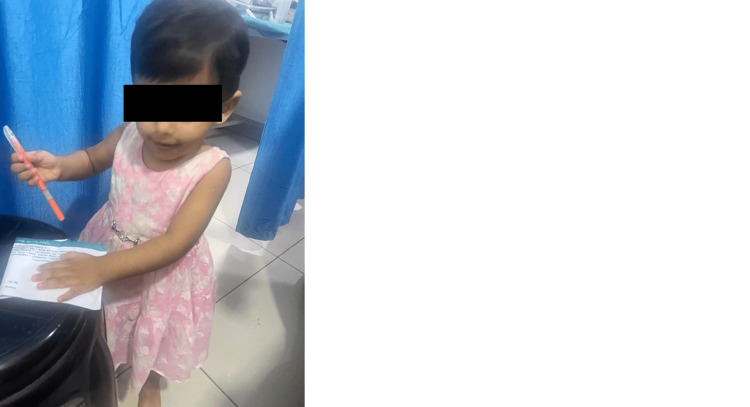
She has been seizure-free for the last 6 months, anti-epileptics have been tapered to just one medication and is able to walk now.

Upon confirmation of diagnosis, the child was initiated on diazoxide at 5 mg/kg/day and prescribed a low-protein diet. Blood sugars increased from 49-63 mg/dl to above 70 mg/dl within 3 days, accompanied by increased activity and responsiveness noted by the mother. To monitor the side effects of diazoxide, investigations were done that revealed normal serum creatinine (0.3 mg/dl), serum uric acid (5.2 mg/dl), complete blood count, and absolute neutrophil count. A normal echocardiography prompted the initiation of hydrochlorothiazide at 1.5 mg/kg/day to prevent fluid overload. Her protein intake was restricted to 1 g/kg/day and a leucine-restricted diet was tried. The child remains under regular follow-up with our multidisciplinary unit, achieving 80% seizure control over a 3-month period. She has been seizure-free in the last 6 months, anti-epileptics have been tapered to just one antiepileptic drug (levetiracetam) and can walk now (Figure 3). Her growth parameters including head circumference have improved on follow-up.

## Discussion

CHI stands as the most common cause of recurrent hypoglycemia in early infancy. Persistent forms of CHI are referred to as persistent hyperinsulinemic hypoglycemia of infancy (PHHI), while transient forms often stem from gestational diabetes, perinatal hypoxia, or intrauterine growth retardation. Despite low blood glucose levels, PHHI is characterized by unsuppressed insulin production [6]. Pathogenic mutations in ABCC8 and KCNJ11 account for nearly half of cases and more than 80% of severe, diazoxide-unresponsive CHI. The next most common cause is HI/HA syndrome [7], initially reported by Cochrane et al. in 1956 [8].

Children with HI/HA syndrome typically exhibit mild hyperammonemia alongside recurrent severe hypoglycemia. Unlike other types of hypoglycemia, HI/HA can also arise in response to protein intake, manifesting as post-prandial hypoglycemia. Children with HI/HA syndrome usually present within the first two years of life and typically have a normal birth weight. However, in rare cases, the condition may not be identified until adulthood [9]. According to one study, the mean age of presentation was found to be 4 months (range: 1 hour of life to 15 months) [10]. Plasma ammonia levels in HI/HA are three to five times higher than normal due to GDH hyperactivity, which increases ammonia release from glutamate and reduces its excretion. Children typically have hyperammonemia without any symptoms; therefore, ammonium-reducing medication (such as sodium benzoate and sodium phenylacetate) is not thought to be helpful in treating HI/HA syndrome [11]. In our case too medications to reduce ammonia were not used. GDH has also been implicated in another form of HI, short-chain-hydroxyacyl-CoA dehydrogenase (SCHAD) deficiency-associated HI.

Two possible causes of seizures are hypoglycemic brain damage and a drop in neurotransmitters such as glutamate and gamma-aminobutyric acid in the brain due to increased GDH. HI/HA is seldom taken into account in the differential diagnosis for hypoglycemia seizures. A critical sample collection during a hypoglycemic episode and a glucagon stimulation test carried out when plasma glucose is less than 50 mg/dl are prerequisites for an accurate diagnosis. One intriguing clinical feature of HI/HA syndrome is the very high incidence of epilepsy. In a group of 16 individuals, 15 had seizures initially, and 43% went on to develop epilepsy [12]. Studies have reported generalized tonic-clonic seizures and absence seizures as the most common types associated with HI/HA syndrome. Another study reported that 79% of HI/HA cases with epilepsy respond to monotherapy, while combination therapy is required in the remaining patients [12,13]. The underlying pathophysiology of the neurodevelopmental manifestations due to the activating mutation of the *GLUD1 *gene is convoluted and not well understood. An interesting feature in our study was episodes mimicking alternating hemiplegia like that of ATP1A2/1A3.

Management consists of a diet low in protein, particularly leucine, and appropriate diazoxide therapy. Despite the positive response to diet control and/or diazoxide, neurological sequelae are not uncommon. Poor outcomes could result from the high cost and restricted availability of diazoxide in our nation [14].

## Conclusions

HI/HA stands out as a rare example of an inborn error of metabolism, wherein mutations in the GLUD1 gene lead to increased enzyme activity. Recognizing this condition is pivotal, especially when a child with a history of hypoglycemia displays elevated ammonia levels on a routine blood sample. Our case presented a diagnostic challenge as the episodes were polymorphic, resembling alternating hemiparesis despite normal EEG and MRI brain, with only one documented hypoglycemic event. In our scenario, hypoglycemia might have been undiagnosed as the sugars were checked during a prolonged paroxysmal episode where the child’s oral intake was inadequate and it is known in the literature that leucine sensitivity after meal produces hypoglycemia in HI/HA. Diazoxide and/or diet typically prove effective for the majority of cases of CHI manifesting beyond the neonatal period. In conclusion, we emphasize the importance of genetic testing in evaluating and treating children with refractory seizures, as reverse phenotyping plays a pivotal role in managing this child.

Timely diagnosis and therapy are paramount in averting the consequences of hypoglycemia, which can lead to irreversible brain damage and impede neurological development.

## References

[1] Martino M, Sartorelli J, Gragnaniello V, Burlina A (2022). Congenital hyperinsulinism in clinical practice: From biochemical pathophysiology to new monitoring techniques. Front Pediatr.

[2] Thornton PS, Stanley CA, De Leon DD (2015). Recommendations from the Pediatric Endocrine Society for Evaluation and Management of Persistent Hypoglycemia in Neonates, Infants, and Children. J Pediatr.

[3] Galcheva S, Demirbilek H, Al-Khawaga S, Hussain K (2019). The genetic and molecular mechanisms of congenital hyperinsulinism. Front Endocrinol.

[4] Hudson RC, Daniel RM (1993). L-glutamate dehydrogenases: Distribution, properties and mechanism. Comp Biochem Physiol B.

[5] Palladino AA, Stanley CA (2010). The hyperinsulinism/hyperammonemia syndrome. Rev Endocr Metab Disord.

[6] Senniappan S, Shanti B, James C, Hussain K (2012). Hyperinsulinaemic hypoglycaemia: Genetic mechanisms, diagnosis and management. J Inherit Metab Dis.

[7] Kane C, Lindley KJ, Johnson PR, James RF, Milla PJ, Aynsley-Green A, Dunne MJ (1997). Therapy for persistent hyperinsulinemic hypoglycemia of infancy. Understanding the responsiveness of beta cells to diazoxide and somatostatin. J Clin Invest.

[8] Cochrane WA, Payne WW, Simpkiss MJ, Woolf LI (1956). Familial hypoglycemia precipitated by amino acids. J Clin Invest.

[9] MacMullen C, Fang J, Hsu BY (2001). Hyperinsulinism/hyperammonemia syndrome in children with regulatory mutations in the inhibitory guanosine triphosphate-binding domain of glutamate dehydrogenase. J Clin Endocrinol Metab.

[10] Stanley CA, Fang J, Kutyna K, Hsu BY, Ming JE, Glaser B, Poncz M (2000). Molecular basis and characterization of the hyperinsulinism/hyperammonemia syndrome: predominance of mutations in exons 11 and 12 of the glutamate dehydrogenase gene. HI/HA Contributing Investigators. Diabetes.

[11] Stanley CA (2004). Hyperinsulinism/hyperammonemia syndrome: Insights into the regulatory role of glutamate dehydrogenase in ammonia metabolism. Mol Genet Metab.

[12] Kapoor RR, Flanagan SE, Fulton P (2009). Hyperinsulinism-hyperammonaemia syndrome: Novel mutations in the GLUD1 gene and genotype-phenotype correlations. Eur J Endocrinol.

[13] Bahi-Buisson N, Roze E, Dionisi C (2008). Neurological aspects of hyperinsulinism-hyperammonaemia syndrome. Dev Med Child Neurol.

[14] Roy K, Satapathy AK, Houhton JA (2019). Congenital hyperinsulinemic hypoglycemia and hyperammonemia due to pathogenic variants in GLUD1. Indian J Pediatr.

